# Co-Encapsulation and In Vitro Gastrointestinal Release of Gallic Acid and Curcumin: A Comparison between Double Emulsions and Templated Core-Laden Bubble Systems

**DOI:** 10.1021/acsomega.6c01415

**Published:** 2026-09-10

**Authors:** Anuj Niroula, Rabia Zia, Mutamed Ayyash, Akmal Nazir

**Affiliations:** † Department of Food Science, College of Agriculture and Veterinary Medicine, 11239 United Arab Emirates University, Al Ain 15551, United Arab Emirates; ‡ Department of Pharmaceutics, Utrecht Institute for Pharmaceutical Sciences, Utrecht University, Utrecht 3584 CG, the Netherlands; § Department of Pharmacology and Therapeutics, College of Medicine and Health Sciences, United Arab Emirates University, Al Ain 15551, United Arab Emirates

## Abstract

This study examines how the intermediate phase in hierarchical colloidal carriers influences the co-encapsulation and in vitro gastrointestinal release of hydrophilic gallic acid and hydrophobic curcumin, using double emulsions (DE; W_1_/O/W_2_) and templated core-laden bubbles (CLBs; W_1_/A/W_2_) as a comparative model system. Monodisperse DEs were prepared by combining high-shear homogenization with T-junction microfluidics. CLBs were obtained by removing the intermediate oil phase through freeze-drying of the parent DEs followed by rehydration, yielding a gaseous intermediate phase. Both systems exhibited high apparent encapsulation efficiencies for gallic acid (>88%) and curcumin (>96%). During storage, DEs showed gradual transfer of gallic acid into the external aqueous phase, whereas CLBs exhibited improved retention, associated with storage in the freeze-dried state and rehydration immediately prior to testing. In vitro digestion experiments showed limited additional release of both compounds under gastric incubation, followed by enhanced release under intestinal conditions. Gallic acid exhibited pronounced intestinal-stage release from both systems, while the CLB workflow maintained a more consistent release profile across storage durations. Curcumin showed predominantly subsequent intestinal-stage release in both systems, with co-encapsulation influencing its release profile. Overall, intermediate-phase state, freeze-drying and rehydration, and dry versus hydrated storage jointly influenced storage retention and intestinal release. Therefore, the paired DE–CLB systems provide a useful comparative formulation platform as hierarchical colloidal carriers rather than as an isolated oil-versus-air experiment.

## Graphical abstract

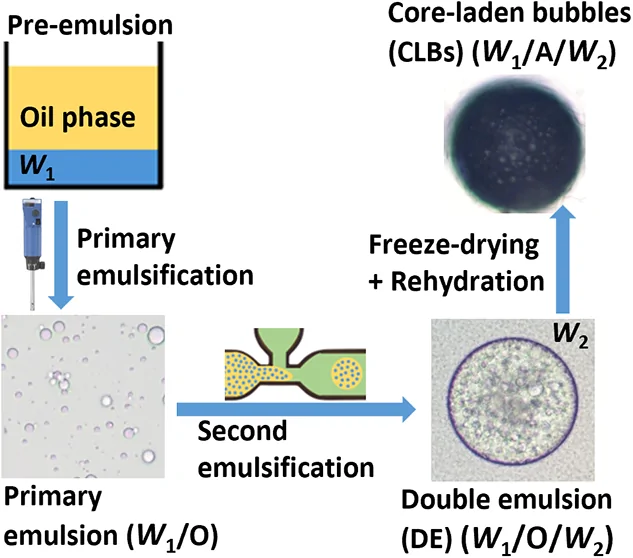

## 1 Introduction

Multiple emulsions, particularly water-in-oil-in-water (W/O/W) systems, have been extensively investigated as delivery platforms for bioactive compounds because of their unique compartmentalized structures, enabling simultaneous incorporation of hydrophilic and lipophilic payloads within distinct phases.^1–3^ In such systems, an internal aqueous phase (W_1_) is dispersed within an intermediate oil phase (O), which is itself dispersed in an external continuous aqueous phase (W_2_), forming a hierarchical structure commonly referred to as a W_1_/O/W_2_ double emulsion (DE).^4,5^ This architecture allows modulation of mass transfer pathways, protection of sensitive compounds, and controlled release during processing and digestion.^6,7^ At the same time, the presence of multiple interfaces introduces challenges related to stability, droplet coalescence, and migration of encapsulated species across phases during storage, particularly under gastrointestinal conditions.^8,9^

Beyond their current use as delivery systems, DEs have also served as versatile templates for generating alternative hierarchical architectures. Over the past two decades, they have been instrumental in fabricating structures such as microspheres, microcapsules and other structured colloidal systems with controlled internal organization.^10–12^ More recently, removal of the intermediate oil phase from W_1_/O/W_2_ DEs by freeze-drying, followed by rehydration, has facilitated the development of liquid-in-air-in-liquid (L/A/L) architectures.^13–15^ These architectures preserve the overall hierarchical organization of their parent DEs while replacing the liquid intermediate phase with a gaseous one. This transformation fundamentally alters phase connectivity, interfacial composition, and transport pathways, and is therefore expected to influence encapsulation efficiency, encapsulation stability during storage, and release behavior under gastrointestinal conditions.^13,16–18^

Since their introduction in 2013,^13^ these templated systems have been widely referred to as “antibubbles” in the literature.^16,17,19–21^ However, unlike classical thin-film antibubbles formed by air entrapment at liquid interfaces,^22–24^ templated systems consist of one or more liquid cores enclosed within a gaseous intermediate phase and stabilized by a particulate or composite outer interface.^13–15^ To reflect this distinct architecture more accurately and to avoid ambiguity with thin-film antibubbles, the present study adopts the term core-laden bubbles (CLBs) to describe DE-templated L/A/L systems. Microfluidic approaches, including T-junction devices, have been widely reported as an effective strategy for producing droplets with narrow size distributions and well-defined internal architectures.^12,25^ Such control is particularly valuable for mechanistic comparisons, as it minimizes variability arising from droplet polydispersity.^26^ The resulting systems share the same precursor formulation and a comparable overall size scale. However, CLB generation additionally involves removal of the volatile oil phase, freeze-drying, dry storage, and rehydration.^19,27^ The paired systems therefore permit examination of the combined effects of intermediate-phase state and processing history rather than the isolated effect of replacing oil with air.

Recent studies have explored the co-encapsulation of hydrophilic polyphenols and hydrophobic bioactive compounds using different systems including DEs, spray-dried systems, and particle-stabilized interfaces, demonstrating the potential of such architectures for co-delivery and, in some formulations, improved retention or bioaccessibility.^28–31^ Despite these advances, most investigations focus on a single delivery system or rely on polydisperse systems, which complicates direct comparison of how colloidal structure governs encapsulation and release. Direct comparisons between DEs and CLB counterparts prepared under controlled and comparable conditions remain limited, particularly for systems designed to co-deliver hydrophilic and hydrophobic bioactives.

In vitro gastrointestinal models provide a controlled framework for probing how colloidal architecture influences structural integrity, encapsulation stability, and release profiles prior to in vivo investigation.^32–34^ Within this context, comparing related architectures (DEs and CLBs) that differ in the intermediate phase state and in the processing steps offers an opportunity to examine the role of phase connectivity and interfacial composition in digestion-responsive delivery. Such comparisons are particularly relevant for co-encapsulation systems, where hydrophilic and hydrophobic payloads may follow distinct release pathways under gastric and intestinal conditions.^29,31,35^

Accordingly, this study investigates the co-encapsulation and in vitro gastrointestinal release behavior of gallic acid, a hydrophilic polyphenol, and curcumin, a hydrophobic bioactive compound, using two related hierarchical colloidal systems: conventional DEs and DE-templated CLBs. The comparison focuses on encapsulation efficiency, storage stability, and digestion-responsive release to assess how transformation of the intermediate phase from liquid oil to air, achieved through freeze-drying and rehydration, influences release behavior. Decane was employed as a volatile intermediate phase to enable controlled templating and CLB formation, serving as a mechanistic model rather than a food-ready formulation. To maintain relevance to practical systems, parallel experiments were conducted using sunflower oil as a non-volatile, food-grade intermediate phase. Because CLB formation requires freeze-drying and rehydration, which may alter interfacial structure and shell properties, the DE and CLB systems differ in both intermediate-phase state and processing history. The study therefore adopts a comparative, model-based approach to examine how phase connectivity and processing collectively influence gastrointestinal release, rather than attributing observed effects solely to phase identity.

## 2 Materials and Methods

### 2.1 Materials

The hydrophobized fumed silica particles, AEROSIL R972 (CAS 68611-44-9) and AEROSIL R816 (CAS 199876-45-4), were kindly provided by Evonik Gulf FZE (Dubai, UAE). Gallic acid monohydrate (CAS 5995-86-8, China), maltodextrin (CAS 9050-36-6, USA), d-mannitol (CAS 69-65-8, China), decane (CAS 124-18-5, Germany), sodium phosphate monobasic monohydrate (CAS 10049-21-5, Germany), pepsin (from porcine gastric mucosa, ≥3200 units/mg protein, CAS 9001-75-6, USA), pancreatin from porcine pancreas (CAS 8049-47-6, China), sodium taurocholate hydrate (CAS 345909-26-4, USA), Folin–Ciocalteu (F–C) reagent (product code 47641, Switzerland), and sodium carbonate (CAS 497-18-8, Germany) were purchased from affiliates of Sigma-Aldrich Chemie GmbH (Darmstadt, Germany). Sodium chloride (CAS 7647-14-5) was purchased from VWR International (Leuven, Belgium). Curcumin (≥98% purity) was obtained from Belle Chemicals (Billings, MT 59104, USA). Isopropanol (CAS 67-63-0) was obtained from Honeywell (Riedel-de Haen, Seelze, Germany). Sunflower oil (SFO) was procured from a local retailer. Distilled water (refractive index (RI): 1.33) was used throughout the study. All formulations were prepared on a wt/wt basis unless specified.

### 2.2 Preparation of Primary Emulsions (PEs), Double Emulsions (DEs), and Core-Laden Bubbles (CLBs)

PEs, DEs and CLBs were prepared as described previously.^19^ High-shear homogenization (HSH) was used to generate PEs, T-junction microfluidics was used to form the DEs, whereas DEs containing a volatile intermediate phase were freeze-dried to obtain freeze-dried double emulsion (FDDE), which were subsequently rehydrated when required to obtain CLBs. The schematic representation of the preparation process of the DE and CLB systems is presented in Figure 1.

**Figure 1:**
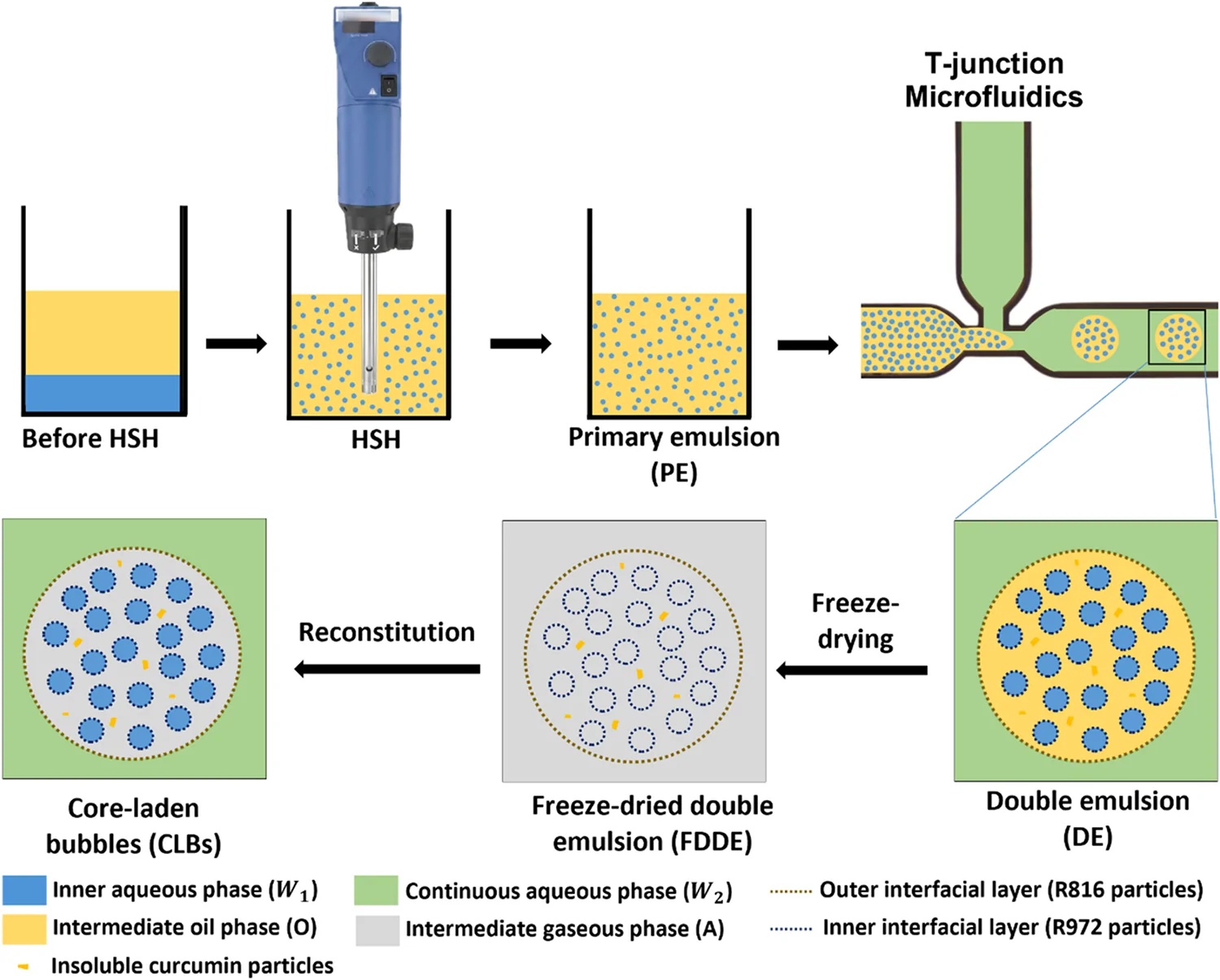
Schematic representation of the preparation workflow for primary emulsions (PEs), double emulsions (DEs), freeze-dried double emulsions (FDDEs), and core-laden bubbles (CLBs).

#### 2.2.1 Preparation of Primary Emulsion (PE) by High-Shear Homogenization (HSH)

PEs were prepared in a 50 mL centrifuge tube by adding 3 mL of W_1_ and 17 mL of oil-phase, and then subjected to HSH using an ULTRA-TURRAX (T25 D with 18 G probe, IKA-Werke GmbH & Co. KG, Staufen, Germany) at 15,000 rpm for 75 s. W_1_ comprised gallic acid (0 or 10 mg/mL) and maltodextrin (10%). The oil phase was prepared by dispersing R972 particles (2.4%) and curcumin (0 or 1 mg/mL) in SFO or decane using a magnetic stirrer (300 rpm, 15 min), followed by 30 s of ultrasonication at 50% amplitude using an ultrasonic processor (750 W, 20 kHz Sonics Vibra-Cell, with CV 334 converter, 13 mm 306-B probe, Sonics and Materials, Inc., Newtown, CT, USA) and used immediately without filtration.

#### 2.2.2 Formation of Double Emulsion (DE) Using Microfluidics

A microfluidic apparatus (The Dolomite Centre Ltd., Royston, U.K.) consisting of a Bambi PT5 compressor (Bambi Air Ltd., Birmingham, U.K.), flow rate sensors (30–1000 μL/min) (The Dolomite Centre Ltd., Royston, U.K.), and an MSZ-AP0-V high-speed digital microscope (Seiwa Optical Co. Ltd.), all controlled via a flow-control software (Dolomite Flow Control Center, version 4.1.9) was used to prepare W_1_/O/W_2_ DEs.

The PE was pumped at a constant flow rate (50 μL/min) through the straight channel of the T-junction microchip (140 μm, 3201045, The Dolomite Centre Ltd., Royston, U.K.), and W_2_ was passed through the perpendicular stem to obtain DE droplets based on a previous study.^19^ The flow of W_2_ was adjusted by pressure control to obtain DE droplets slightly smaller than the microchannel diameter (i.e., <140 μm). The final PE/*W*_2_ proportion of 1:25 was maintained by adding the calculated amount of W_2_ to the collected DEs to ensure uniformity among the samples. All samples were collected in a volume of 1 mL in 5 mL Eppendorf tubes.

Before emulsification, the flow-rate sensors were calibrated by passing the PEs and W_2_ at different known flow rates. W_2_ was prepared by dispersing R816 (3%) with maltodextrin (10%) and mannitol (10%) using a magnetic stirrer (300 rpm, 15 min), followed by 30 s of ultrasonication at 50% amplitude using the same ultrasonic processor. Maltodextrin and mannitol were included as cryoprotective and osmotic agents based on previous studies.^13,18,21^

#### 2.2.3 Freeze-Drying and Rehydration to Obtain Core-Laden Bubbles (CLBs)

The DEs with decane as the intermediate phase were frozen at −80 ± 3 °C (Ultra-freezer, BINDER GmbH, Germany) and lyophilized using a freeze dryer (LyoAlfa 15, Telstar, Spain) at 0.01 mbar vacuum and condenser temperature of −83 ± 3 °C for 24 h to obtain FDDEs. FDDEs were rehydrated to their original volume by adding distilled water, resulting in the formation of CLBs.

#### 2.2.4 Storage Conditions

For storage, 1 mL samples were collected in 5 mL centrifuge tubes and stored at 4 ± 2 °C. FDDEs generated by freeze-drying the parent DE were stored in the dry (lyophilized) state at the same temperature and were rehydrated to form CLBs immediately before analysis. Consequently, comparisons of storage retention reflect both carrier architecture and processing/storage history.

### 2.3 Microscopic Imaging

Optical and fluorescence images were captured with a Delphi-X Observer fluorescence microscope (Euromex, Arnhem, The Netherlands) fitted with 10×, 20×, and 100× objective lenses. PEs were diluted five times with their continuous phase solvent and examined on a cavity slide. Image capture and analysis were performed using Euromex ImageFocus α software (version 1.3.7.12967.20180920). DEs were imaged without dilution. CLBs were imaged 10 min after rehydration unless otherwise stated.

### 2.4 Size, Dispersity, and Rehydration Coefficient (RC)

The diameters of PE (*D*_PE_), DE (*D*_DE_), and CLBs (*D*_CLB_) were determined from microscopic images using *ImageJ* software (version 1.54 g).^36^ Images at 100× magnification were used for PEs, whereas images at 20× magnification were employed for DEs and CLBs. For each sample, 3 batches were evaluated, each comprising at least 100 droplets.

For the non-spherical and partially coalesced CLBs, the equivalent circular diameter (ECD) was calculated as

1 $$ECD=2\times \sqrt{A/\pi }$$

where, *A* is the projected area of the CLB.

The size dispersity was evaluated as the coefficient of variation (CV) calculated as

2 CV=σ/μ

where σ is the standard deviation and μ is the arithmetic mean droplet diameter. The terms CV_PE_, CV_DE_, and CV_CLB_ denote CV values for PEs, DEs, and CLBs, respectively. For emulsion systems, CV values of <25% are often considered indicative of relatively narrow size distributions in conventional emulsification processes. However, studies employing microfluidic fabrication typically apply a more stringent criterion, defining monodispersity as a CV of <10 %.^19,37^ Accordingly, a CV < 10% was adopted in this study as the threshold for monodispersity.

The RC was used to assess the ability of parent DE droplets to recover their original size upon rehydration. Because the intermediate liquid phase is replaced by air during CLB formation, RC was calculated by comparing the diameter after rehydration (*D*_CLB_) to the diameter before freeze-drying (*D*_DE_)­

3 $$RC=D_{\mathrm{CLB}}/D_{\mathrm{DE}}$$

The RC value of 1 indicates *D*_CLB_ = *D*_DE_; values <1 indicate shrinkage (*D*_CLB_ < *D*_DE_), and values >1 indicate swelling (*D*_CLB_ > *D*_DE_). To minimize the influence of droplet coalescence and CLB rupture on RC determination, only non-coalesced DEs and CLBs were included to analyze RC.

### 2.5 Encapsulation Efficiency (EE), Encapsulation Stability (ES), and Gastrointestinal Release

#### 2.5.1 Sample Collection, Storage, and Preparation

Separate sample tubes comprising of 1 mL sample were used for each storage time point. Evaluations were performed both immediately and after 7, 14, and 21 days of storage at 4 ± 2 °C. For CLB preparation, DEs were frozen, lyophilized and stored as described in Sections 2.2.3 and 2.2.4. Prior to evaluation, FDDEs were rehydrated to their original volume with distilled water. The tubes were gently inverted five times to ensure uniform mixing and then allowed to stand for at least 5 min.

#### 2.5.2 Encapsulation Efficiency (EE) and Change during Storage

EE was defined as the percentage of total recoverable analyte that was not detected in the isolated *W*_2_. To 1 mL of each sample (DEs or CLBs), 4 mL of distilled water was added and gently mixed, followed by centrifugation at 5000*g* for 5 min. Approximately 0.45 mL was withdrawn from the middle aqueous layer using a sterile 5 mL syringe, avoiding the creamed top layer and sediment. From this, 0.3 mL (*V*_B_) was transferred to a 2 mL graduated Eppendorf tube, while the remaining sample was returned to the original tube. An equal volume of isopropanol (0.3 mL) was added and vortexed for 2 min, then centrifuged at 12,000*g* for 5 min. The remaining 4.7 mL (*V*_A_) of original diluted sample was then vortexed to mix evenly. A 0.6 mL aliquot was drawn and mixed with equal amount of isopropanol (0.6 mL), vortexed, and used for analyte quantification. EE and release into *W*_2_ were calculated as follows

4 $$EE=\left(1-C_{B}/C_{T}\right)\times 100\%$$

5 $$r\mathrm{elease in isolated fraction}=\left(C_{B}/C_{T}\right)\times 100\%$$

where *C*_B_and *C*_T_ are concentrations measured in the isolated W_2_ fraction and the volume-weighted total recoverable analyte concentration in DE (or CLB) respectively. *C*_T_ was calculated as

6 $$C_{T}=\left(C_{B}\times V_{B}+C_{A}\times V_{A}\right)/\left(V_{B}+V_{A}\right)$$

where *C*_A_ is the analyte concentration measured in the remaining sample after removal of isolated *W*_2_.

#### 2.5.3 Static Simulated Gastrointestinal Study

Separate samples were evaluated in the gastrointestinal study after 48 h and 14 days of storage. Simulated gastrointestinal fluids were prepared using a modified static digestion approach based on previous studies^38–40^ with the compositions and pH conditions specified below. Simulated gastric fluid (SGF) was prepared as an aqueous solution containing pepsin (2 mg/mL) and NaCl (2 mg/mL), with the pH adjusted to 1.8 using 1 M HCl. Simulated intestinal fluid (SIF) consisted of an aqueous solution containing monobasic sodium phosphate (6.8 mg/mL), pancreatin (2 mg/mL), and sodium taurocholate (NaTC) (15 mM), with the pH adjusted to 6 using 5 M NaOH.

The temperature was maintained at 37 ± 1 °C throughout the digestion period. To simulate gastric conditions, 10 mL of preheated SGF (37 ± 1 °C) was added to 1 mL of each sample (DEs or CLBs). Aliquots (0.3 mL) were collected at 0 h and after 2 h of gastric incubation. After 2 h, 10 mL of SIF was added, and the pH was adjusted to 6.0 using 1 M NaOH. The 2 h time point was therefore the initiation of the intestinal stage. Additional aliquots (0.3 mL) were collected at 2.25, 4, and 6 h to quantify intestinal release. To exclude intact DE or CLB, 1 mL of sample was drawn from the middle of the aqueous fluid, then centrifuged at 5000*g* for 5 min. An aliquots (0.3 mL) were withdrawn avoiding the creamed top layer and sediment with a 5 mL sterile syringe in a 2 mL Eppendorf and the remaining sample was returned to the original tube. An equal volume of isopropanol was added to each aliquot to solubilize curcumin and prepare a 50% (v/v) isopropanol solution. For volume-weighted total recovered concentration (*C*_t_), isopropanol (half the volume of the remaining sample) was added, vortexed for 2 min, and centrifuged at 12,000*g* for 5 min. To 0.6 mL of the supernatant, 0.2 mL of isopropanol was added to maintain a 50% (v/v) isopropanol concentration. *C*_t_ was calculated as

$$C_{t}=\left(C_{b0}+C_{b2}+C_{b.25}+C_{b4}+C_{b6}\right)\times (V_{b}+C_{a}\times V_{a})/\left({5\times V}_{b}+V_{a}\right)$$ 7

where *C*_b0_, *C*_b2_, *C*_b2.25_, *C*_b4_, and *C*_b6_ are the dilution-corrected concentrations in samples drawn successively during the study, *C*_a_ is the dilution-corrected concentration in the sample evaluated after disruption with isopropanol, *V*_b_ is the sample drawn to evaluate before disruption, and *V*_a_ is the volume of sample used for interfacial disruption. In this study, a constant value of 300 μL was used for *V*_b_.

Release was quantified as pre-release (0 h), gastric release (0–2 h), intestinal triggered release (2–2.25 h), and subsequent intestinal-stage release (2.25–6 h). This design captures stimulus-induced release events rather than continuous diffusion kinetics.

#### 2.5.4 Determination of Curcumin Content

For curcumin quantification, 0.25 mL aliquots were pipetted into a 96-well plate, and the absorbance was measured at 425 nm using a UV–vis spectrophotometer (Thermo Multiskan GO Microplate Reader, Finland). Samples were diluted with 50% (v/v) isopropanol in water when necessary. Distilled water with the same SGF and SIF matrix was used as a blank sample. Concentrations were determined using a calibration curve prepared with curcumin standards ranging from 0 to 0.01 mg/mL in 50% (v/v) isopropanol.

#### 2.5.5 Determination of Gallic Acid Content

Gallic acid content was estimated using the Folin–Ciocalteu (F–C) method with minor modifications as described previously.^41^ Briefly, 0.25 mL of the sample was mixed with 0.25 mL of 0.2 M F–C reagent and gently mixed for 5 min. Then, 0.25 mL of sodium carbonate (7.5% w/v) was added and thoroughly mixed. The mixture was incubated at 45 ± 2 °C for 45 min. After incubation, 0.3 mL was pipetted to a 96-well plate, and absorbance was measured at 765 nm. Distilled water with same SGF and SIF matrix was used as a blank sample. Total polyphenol content was expressed as gallic acid equivalent (GAE), using a calibration curve (*R* ^2^ ≥ 0.98) prepared with gallic acid standards (0–0.02 mg/mL).

For samples containing both gallic acid and curcumin, the contribution of curcumin to the F–C response was estimated using a separately prepared calibration curve (*R* ^2^ ≥ 0.98) with curcumin standards (0–0.01 mg/mL) and subtracted from the total polyphenol content to obtain an estimate of gallic acid content.

### 2.6 Statistical Analysis

All statistical analyses and graphical representations were performed using JMP Pro 15.0.0 (SAS Institute Inc., Cary, NC, USA). Experiments were conducted in triplicate, and results are reported as mean ± standard deviation. One-way analysis of variance (ANOVA) followed by Tukey’s honestly significant difference (HSD) test was used for comparisons among individual treatment means. Storage-dependent EE was analyzed separately for gallic acid and curcumin using analysis of covariance (ANCOVA), with system type (DE or CLB) and loading mode (individual encapsulation or co-encapsulation) as categorical factors and storage time as a continuous covariate. Each gastrointestinal release fraction was analyzed separately for each compound using factorial ANOVA, with system type, storage duration, and loading mode as fixed factors. Non-significant terms were removed while preserving model hierarchy. Statistical significance was accepted at *p* < 0.05.

## 3 Results and Discussion

### 3.1 Primary Emulsions (PEs)

All PEs were polydisperse with *D*_PE_ in the range of 2.77–2.97 μm and CV_PE_ of 68–75% (Figure 2). Mean droplet diameter did not differ significantly among formulations (*p* > 0.05). Micrographs revealed a greater number of visible undissolved curcumin particles in decane (Figure 2b,c) than in SFO (Figure 2e,f). Because phase-specific curcumin solubility was not measured, this observation is interpreted as a difference in apparent dispersion state.

**Figure 2:**
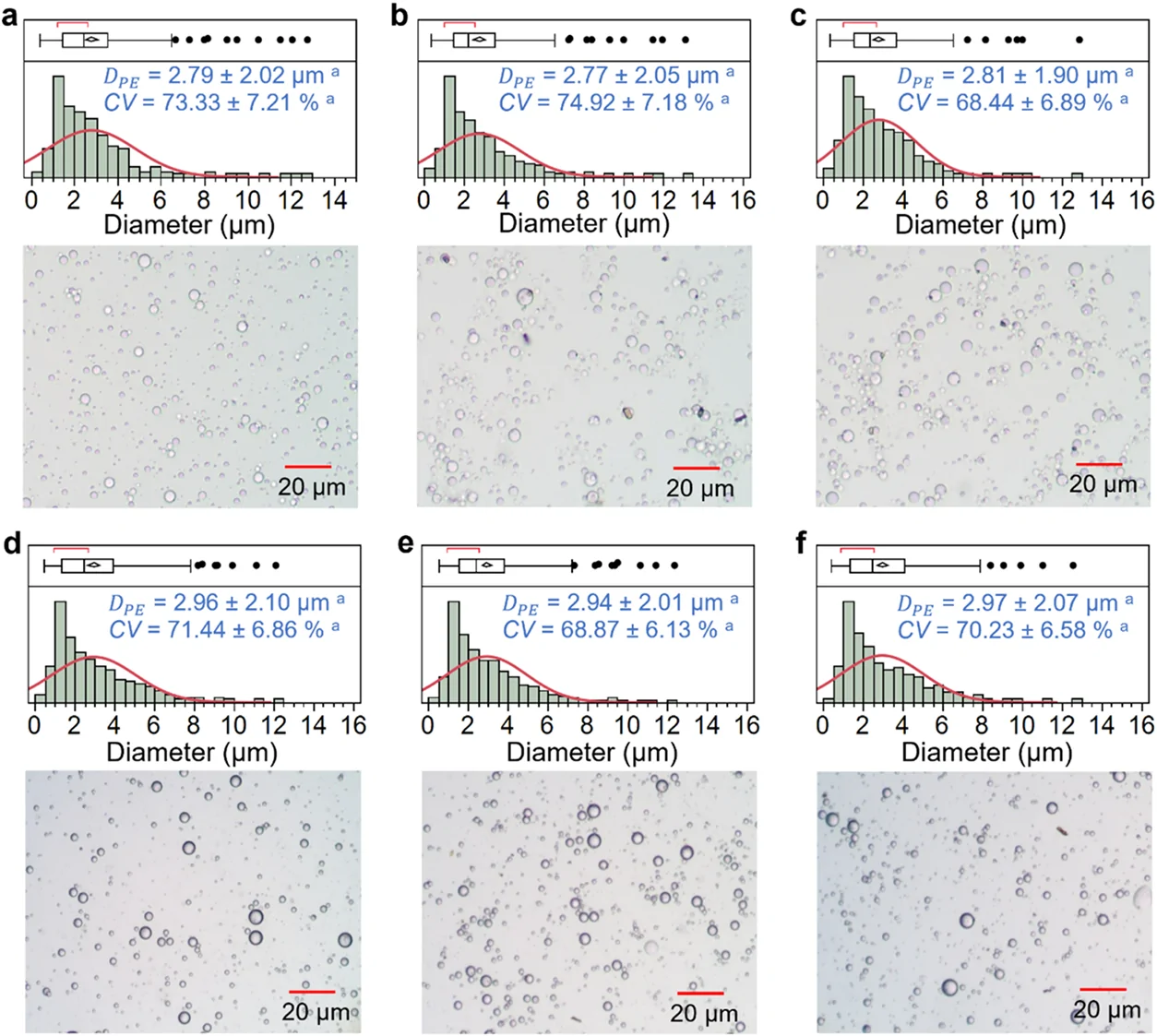
Optical micrographs and corresponding droplet size distributions of primary emulsions (PEs). (a–c): inner aqueous phase (W_1_) dispersed in decane, (d–f): W_1_ dispersed in sunflower oil, (a, d): 10 mg/mL gallic acid in W_1_, (b, e): 1 mg/mL curcumin in the oil phase, and (c, f): 10 mg/mL gallic acid in W_1_ and 1 mg/mL curcumin in the oil phase.

### 3.2 Double Emulsions (DEs)

Microfluidic secondary emulsification produced DE droplets with *D*_DE_ of 131.85–132.01 μm and CV_DE_ of 2.6–3.32% (Figure 3). These values satisfied the adopted monodispersity criterion of CV < 10%. This narrow size distribution minimizes the influence of droplet size on EE and release behavior of the active components,^26,42^ but does not eliminate effects arising from composition and processing. Visible curcumin particles persisted in decane-based DEs; consequently, apparent curcumin EE may include carrier-associated particulate curcumin rather than exclusively molecularly dissolved curcumin.

**Figure 3:**
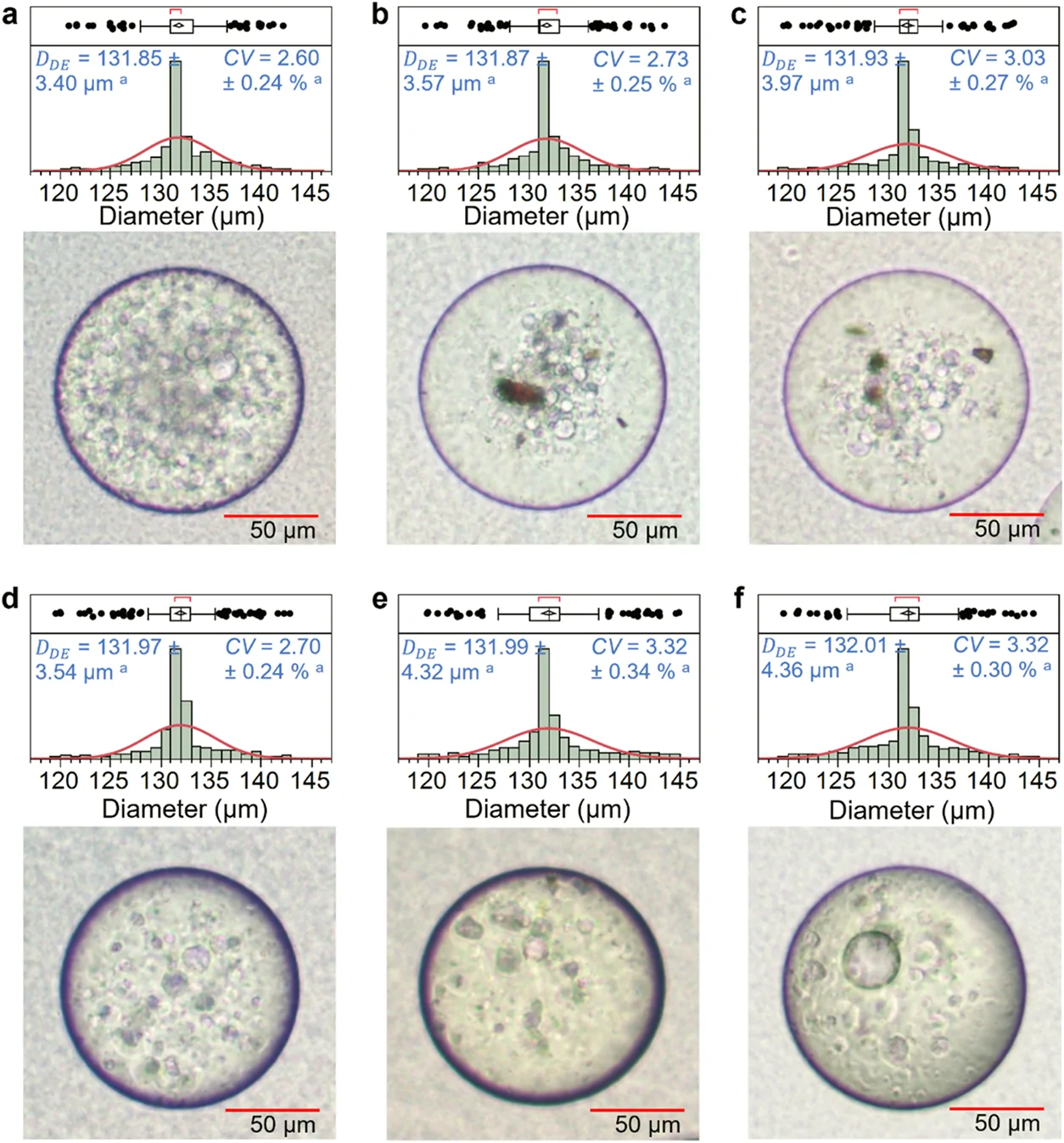
Optical micrographs and corresponding droplet size distributions of double emulsions (DEs). (a–c): inner phase (W_1_) dispersed in decane, (d–f): W_1_ dispersed in sunflower oil, (a, d): 10 mg/mL gallic acid in W_1_, (b, e): 1 mg/mL curcumin in the oil phase, and (c, f): 10 mg/mL gallic acid in W_1_ and 1 mg/mL curcumin in the oil phase.

### 3.3 Core-Laden Bubbles (CLBs)

Freeze-drying of decane-based DEs removed the solvents (volatile intermediate phase and water) present in the system, resulting in dry FDDEs. These FDDEs formed CLBs upon rehydration. SFO-based DEs were not converted to CLBs because SFO was non-volatile under the applied freeze-drying conditions. Therefore, the direct parent-templated comparison was restricted to decane-based DEs and their corresponding CLBs.

#### 3.3.1 Rehydration of Freeze-Dried Double Emulsion (FDDE)

When required, FDDEs were rehydrated to their original volume using distilled water, thereby immediately restoring W_2_. During microscopic observation, the initially opaque FDDE structures progressively became more optically distinguishable, revealing internal aqueous droplets surrounded by a dark intermediate region (Figures 4 and 5). Based on optical contrast and morphology reported for comparable DE-templated systems, this intermediate region was interpreted as a gaseous phase. The reconstitution is interpreted to occur due to water vapor pressure gradient arising from the low water activity of the dried internal cores. Water vapor diffuses through the intermediate region and condenses within the hygroscopic maltodextrin-containing cores, thereby reconstituting W_1_, while the intermediate phase remains gaseous, resulting in the formation of W_1_/A/W_2_ CLBs.^13,14^ Similar rehydration mechanisms and W_1_/A/W_2_ architectures have been reported in several previous studies,^16–18,21^ with some systems maintaining the configuration for at least 2 weeks.^21^ In the present study, rehydration produced a heterogeneous population comprising intact CLBs (Figure 4a), along with partially coalesced structures (Figure 4b), and some ruptured structures that released W_1_ into W_2_ (Figure 4c–d).

**Figure 4:**
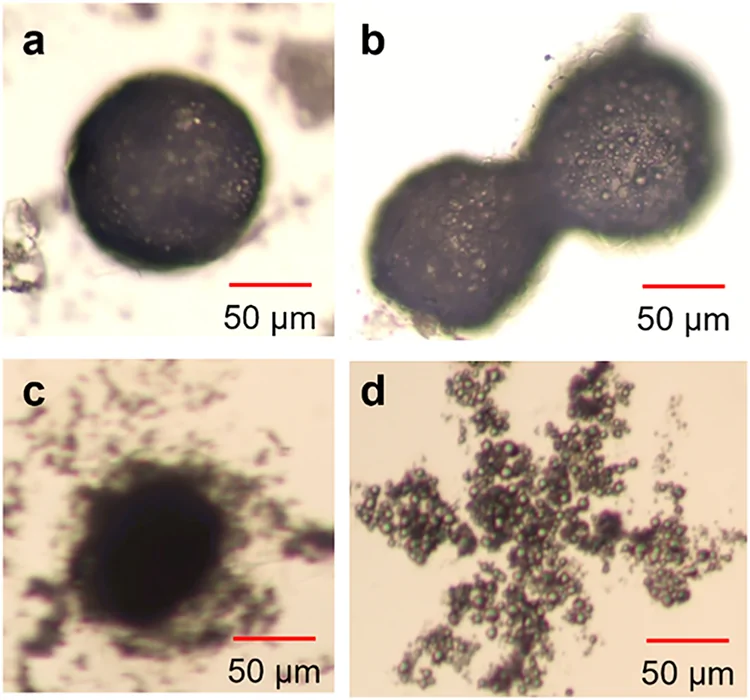
Representative optical micrographs obtained after rehydration of freeze-dried double emulsions (FDDEs). (a): structurally intact core-laden bubbles (CLBs), (b): partially coalesced CLBs, (c, d): ruptured structures showing release of the internal aqueous core (W_1_) into the continuous aqueous phase (W_2_).

**Figure 5:**
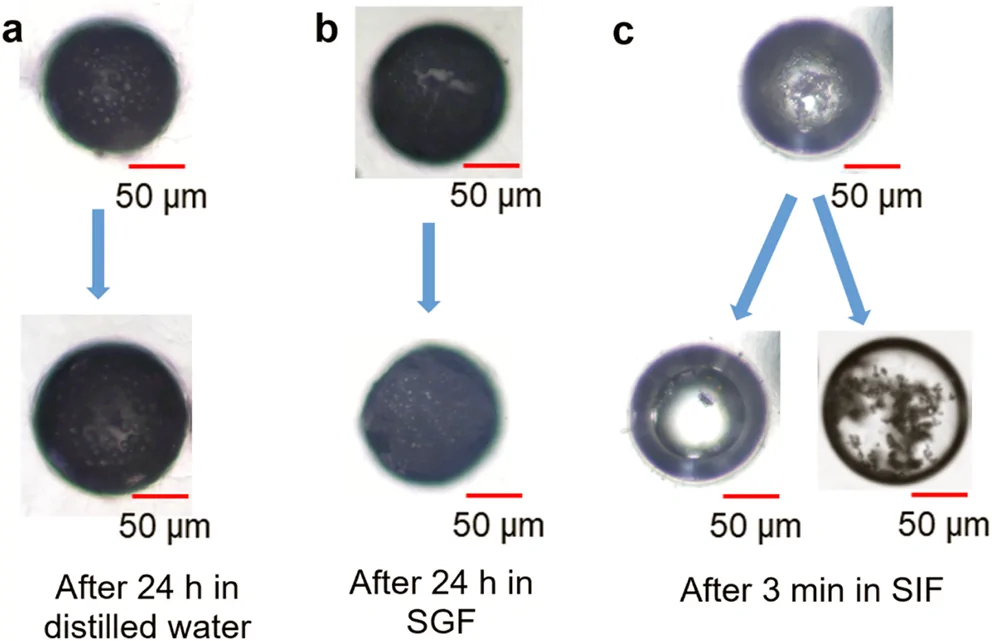
Representative optical micrographs illustrating the morphological changes in core-laden bubbles (CLBs) following exposure to (a) distilled water, (b) simulated gastric fluid (SGF), and (c) simulated intestinal fluid (SIF). The upper images correspond to the first 10 min of rehydration before introduction of SGF and SIF.

#### 3.3.2 Size and Dispersity

At 10 min after rehydration, the intact, non-coalesced CLB sub-population exhibited a *D*_CLB_ of 123.93 ± 10.98 μm, with a CV_CLB_ of 8.81 ± 0.88%. The corresponding RC was 0.92 ± 0.03. Because coalesced and ruptured structures were excluded, these values describe the intact CLB subpopulation rather than the complete rehydrated sample. The lower initial RC may reflect incomplete rehydration, freeze-drying-induced shrinkage, or both. Ideally, freeze-drying should preserve structural integrity; however, shrinkage or collapse can occur in systems with high moisture content and low surface-area-to-volume ratios.^19,43^ Comparable size reductions immediately after rehydration have been reported for multicore DE-templated CLBs.^16–19,21^

Between 10 min and 24 h after rehydration in distilled water, *D*_CLB_ increased to 139.57 ± 10.38 μm with CV_CLB_ of 7.43 ± 0.92 %, and RC increased to 1.06 ± 0.03, indicating continued structural and osmotic equilibration after rehydration. An increase in size was also reported in a proof-of-concept study involving core-shelled CLBs,^14^ in which a thick hydrophobic silica layer at the outer interface was proposed to retain the gaseous intermediate phase while permitting inward diffusion of water vapor. As a result, the CLB size was greater than expected from liquid-state W_1_ droplets alone. Despite some partial coalescence, CV_CLB_ remained below 10 % at both measurement times, confirming a narrow size distribution within the analyzed intact CLB subpopulation.

#### 3.3.3 Stability of Core-Laden Bubbles (CLBs)

CLBs rehydrated in distilled water or exposed to SGF retained their overall morphology during the 24 h observation period, whereas exposure to SIF induced rapid structural destabilization (Figure 5). In distilled water (Figure 5a), CLBs initially increased in size during the first 24 h, likely due to osmotic water uptake into the core during post-rehydration equilibration and subsequently maintained their size and integrity. When SGF was added after 10 min of rehydration (Figure 5b), CLBs decreased in size over the 24 h observation period. This shrinkage may reflect water transfer from W_1_ to W_2_ in response to the ionic strength and low pH of SGF, thereby decreasing the internal aqueous phase volume. In addition, reduced electrostatic repulsion between particles at the outer interface under acidic conditions may promote closer particle packing and contribute to a more compact overall structure.^2^ Additionally, gas loss through interfacial defects may also contribute to the observed size reduction; however, gas transport was not quantified in the present study. Unlike surfactant-stabilized interfaces, particle-stabilized interfaces can remain structurally intact despite the presence of nanopores or minor interfacial defects, owing to the firm anchoring of particles at the interface. Consequently, such defects do not necessarily result in immediate collapse unless substantial particle displacement, desorption or interfacial disruption occurs.^2,14,18,44^

In contrast, exposure to SIF led to visible destabilization within 3 min (Figure 5c). Initially, W_1_ droplets were expelled from the gaseous intermediate phase, leaving behind air-in-liquid (A/L) bubbles. This expulsion may have been promoted by NaTC, a surface-active bile salt present in SIF. NaTC may alter the silica-stabilized interface through its surface activity and interactions with silica or other interfacial species, thereby contributing to interfacial weakening.^45–47^ Such interactions could facilitate local particle displacement or the formation of uncovered interfacial regions through which the internal aqueous droplets escape. The higher pH of SIF may also modify silica surface charge and interparticle interactions, although this effect was not independently evaluated in the present study. Thus, the observed destabilization likely reflects the combined effects of bile salts, pH, ionic composition, osmotic imbalance, and interfacial restructuring rather than the action of a single component.

The gaseous intermediate phase may further facilitate outward movement of W_1_ droplets because it provides substantially less viscous resistance than a liquid-oil intermediate phase. As destabilization progressed, penetration of W_2_ into the gaseous region produced water-filled multicompartment remnants, morphologically consistent with water-in-water-in-water (W/W/W)-like arrangements with residual W_1_ droplets (Figure 5c). Their formation may be associated with NaTC adsorption on the silica-stabilized interface, which has previously been reported to alter particle wettability and destabilize particle-dominated interfaces.^45,47^ Changes in interparticle interactions at the intestinal pH may additionally increase interface permeability and promote penetration of the continuous aqueous phase.

It should be noted that some disrupted or W/W/W-like structures may already arise during rehydration, regardless of hydration medium, because of damage to the interfacial layers during freezing and freeze-drying. Structures with extensively disrupted interfaces may release W_1_ immediately upon rehydration and therefore fail to form intact CLBs. Such structures represent rehydration defects rather than destabilization of initially intact CLBs under gastrointestinal conditions. However, partially disrupted structures that persist after rehydration may still contribute to the observed destabilization behaviors in SIF. Because bile-salt adsorption, silica-particle displacement, gas loss, interfacial permeability, and aqueous-phase transport were not directly quantified, the proposed mechanisms should be regarded as interpretations consistent with the observed morphology and previous studies rather than as independently demonstrated mechanisms.

### 3.4 Encapsulation Efficiency (EE) and Change during Storage

EE (%) of gallic acid and curcumin in DEs and CLBs during storage for up to 3 weeks at 4 ± 2 °C is presented in Figure 6. ANCOVA showed that the storage profiles of gallic acid differed significantly between DEs and CLBs (*p* < 0.05). The influence of co-encapsulation on gallic acid retention also differed between the two systems, although co-encapsulation did not produce a significant overall effect when both systems were considered together (*p* > 0.05). For curcumin, small but significant storage-dependent differences were observed between DEs and CLBs (*p* < 0.05). No significant lack of fit was detected for either model. The following subsections therefore focus on the magnitude and practical relevance of the formulation-specific differences.

**Figure 6:**
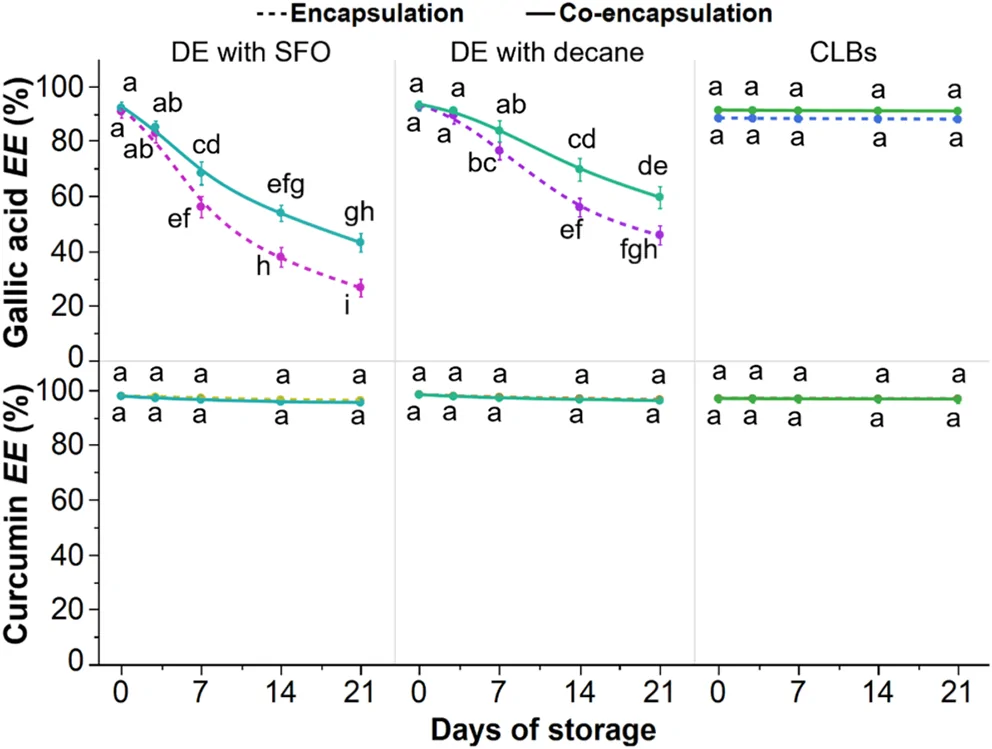
Encapsulation efficiency (EE, %) of gallic acid and curcumin, encapsulated individually and co-encapsulated in double emulsion (DE) and core-laden bubbles (CLBs) during storage for up to 3 weeks at 4 ± 2 °C. The error bars represent the standard deviation. Different lowercase letters indicate significant differences among the individual sample means (*p* < 0.05) for each active component. Color coding is used solely to aid visual comparison.

#### 3.4.1 Gallic Acid

The initial EE of gallic acid in DEs was in the range of 90–93% (Figure 6), with no significant difference between SFO- and decane-based DEs at day 0. These high initial values are consistent with the controlled secondary emulsification achieved using microfluidic emulsification, which limited the immediate escape of W_1_ droplets into W_2_ during DE formation. Unlike conventional two-step homogenization, which often leads to droplet coalescence and escape of actives or entire W_1_ droplets into W_2_,^48^ microfluidic emulsification enables more controlled droplet formation, enhancing EE.^25,49^

During 21 days of storage, the EE decreased significantly in both DE formulations (*p* < 0.05). This decrease indicates progressive transfer of gallic acid from W_1_ into the external aqueous phase during storage. This transfer may occur through molecular transport across the intermediate oil phase, transport associated with internal aqueous droplets, or localized disruption of the interfaces separating W_1_ and W_2_.^8^ However, on day 21, EE in decane-based DE (45.87 ± 3.51 %) was significantly higher (*p* < 0.05) than SFO-based DE (26.73 ± 3.25 %). Because the formulations differed only in the intermediate oil phase, the difference in gallic acid retention reflects the effect of oil-phase properties like polarity, analyte partitioning, and permeability,. Besides molecular transport, the release of gallic acid from W_1_ to W_2_ may occur via physical escape of W_1_ droplets from the intermediate oil phase, followed either by their merging with W_2_ or by persistence as dispersed W_1_ droplets in W_2_.^2,50^ This mechanism has been reported previously for DEs;^34,51^ however the contribution of such entire-droplet escape was not quantified in this study.

In contrast to a previous study, where significant reduction of EE (from ∼98 % to ∼90%) (*p* < 0.05) was observed during freeze-drying and rehydration of DEs,^19^ the EE of CLBs was 88.42 ± 1.01%, only slightly lower than the corresponding DE (92.63 ± 1.28%). This marginal reduction was consistent with observed ruptured structures (Figure 4c,d) releasing W_1_ into W_2_. No statistically detectable change (*p* > 0.05) in the gallic acid EE was detected in the FDDE/CLB workflow during the three-week storage period. This is consistent with storage of the CLB precursors in the freeze-dried state, in which the absence of continuous liquid phases limits molecular mobility and phase-to-phase transfer before rehydration.^19^ However, this comparison should not be interpreted as evidence that a gaseous intermediate phase is intrinsically more stable than an oil phase. Besides intermediate phase, the inner and outer phases of DEs were aqueous, whereas CLBs were stored in dry solid precursor form i.e., FDDEs. The observed difference therefore reflects the combined effects of freeze-drying, dry storage, rehydration, and formation of the gaseous intermediate phase. Accordingly, the results demonstrate improved gallic acid retention by the complete FDDE/CLB storage workflow over the three-week storage period rather than an isolated stabilizing effect of air as the intermediate phase.

#### 3.4.2 Curcumin

Curcumin maintained a high EE (>96%) in all formulations throughout the three-week storage period (Figure 6). This behavior may be associated with the very low water solubility of curcumin, which limits its migration from the oil phase to W_2_.^2^ It should be noted that visible undissolved curcumin particles were present in the decane-based PEs and remained observable in the corresponding DEs. Consequently, the measured apparent EE represents retention of total isopropanol-recoverable curcumin within the carrier-associated fraction and does not necessarily indicate complete molecular dissolution or molecular encapsulation. The high retention is consistent with the very low aqueous solubility of curcumin, which limits its transfer into W_2_.^52^

#### 3.4.3 Co-Encapsulation

Co-encapsulation did not significantly alter the initial apparent EE of either compound. During storage, however, its effect on gallic acid retention depended on the system. In DEs, co-encapsulation reduced the loss of gallic acid during storage (Figure 6). Co-encapsulation resulted in significantly higher (*p* < 0.05) EE of gallic acid on day 7 for SFO-based DE and on day 14 for decane-based DE. By day 21, co-encapsulation resulted in a significant increase (*p* < 0.05) in retention of gallic acid, from 26.73 ± 3.25 % to 43.15 ± 3.39 % in SFO-based DE and from 45.87 ± 3.51 % to 59.68 ± 3.99 % in decane-based DE. This increased retention indicates that curcumin co-encapsulation influenced gallic acid retention in the DEs. Although the mechanism responsible for the improved retention was not resolved, the formulations differed only in the presence of the second active compound. The observed effect may therefore reflect co-encapsulation-dependent changes in analyte partitioning, dispersion state, interfacial organization, or internal-droplet transport. Co-encapsulation-dependent changes in retention and release have also been reported in other delivery matrices.^28,53^ However, no direct evidence of gallic acid–curcumin complexation was obtained in the present system. In contrast, co-encapsulation had no significant effect (*p* > 0.05) on the retention of gallic acid in CLBs. This lack of an observable co-encapsulation effect may reflect the already limited molecular mobility and phase-to-phase transport during storage of the freeze-dried precursors of CLBs as discussed in Section 3.4.1. Co-encapsulation did not produce significant differences (*p* > 0.05) in curcumin EE for any formulation at all storage times, and all values remained above 95%.

Overall, co-encapsulation enhanced gallic acid retention in DEs, while apparent gallic acid EE in the FDDE/CLB workflow remained unchanged regardless of loading mode.

### 3.5 In Vitro Digestion and Gastrointestinal Release

The in vitro gastrointestinal release study was conducted on DEs and CLBs after 48 h and 14 days of storage at 4 ± 2 °C (Figure 7). For gallic acid, the extent of pre-release and gastric-stage release changed with storage duration, and these changes differed between DEs and CLBs (*p* < 0.05). SIF-triggered release also varied with storage duration, loading mode, and system type, with significant interactions among these factors; however, this model showed significant lack of fit and is therefore interpreted cautiously. Subsequent intestinal-stage release differed between individually encapsulated and co-encapsulated samples, between DEs and CLBs, and between the two storage durations, with the effect of storage duration differing between the two systems (*p* < 0.05). The unreleased gallic acid fraction also differed with storage duration and loading mode (*p* < 0.05). For curcumin, pre-release did not differ significantly among the studied conditions. Gastric-stage release differed between loading modes, whereas SIF-triggered release differed between DEs and CLBs (*p* < 0.05). Subsequent intestinal-stage release differed between systems and loading modes, and the effect of loading mode was more pronounced in CLBs than in DEs (*p* < 0.05). The unreleased curcumin fraction likewise differed between DEs and CLBs and between loading modes (*p* < 0.05). No significant lack of fit was detected for the remaining models. The following subsections therefore focus on the magnitude and practical relevance of the formulation-specific differences.

**Figure 7:**
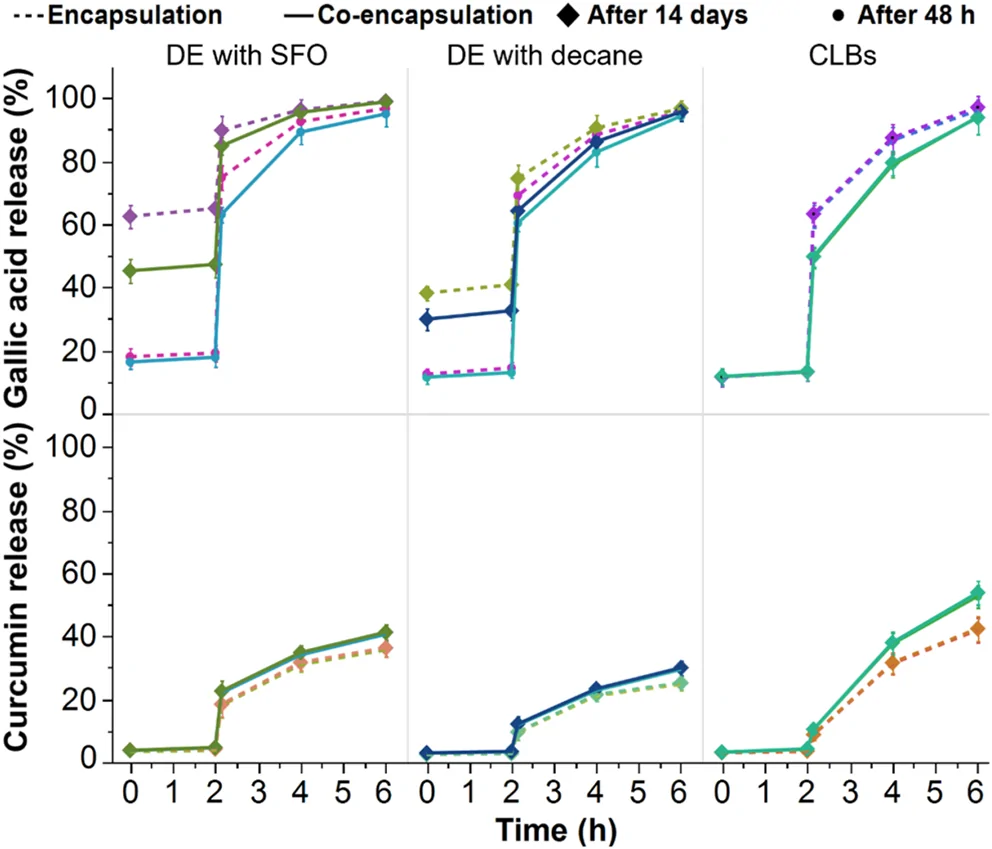
Phase-resolved in vitro gastrointestinal release (%) of gallic acid and curcumin, encapsulated individually and co-encapsulated in double emulsions (DEs) and core-laden bubbles (CLBs) after 48 h and 14 days of storage at 4 ± 2 °C. The error bars represent the standard deviation. Color coding is used solely for visual clarity. Statistical differences are provided in Supporting Tables S1 and S2.

#### 3.5.1 Gallic Acid

At the onset of the gastric stage, the pre-released gallic acid fraction was significantly higher (*p* < 0.05) in DEs stored for 14 days than in those stored for 48 h. For SFO-based DEs, this fraction increased significantly from 18.02 ± 2.78% to 62.47 ± 3.63%, while for decane-based DE, it increased significantly from 12.48 ± 1.76% to 38.20 ± 2.19% (*p* < 0.05; Supporting Table S1). In contrast, the pre-released fraction of gallic acid in CLBs remained essentially unchanged, at 11.52 ± 2.40% after 48 h and 11.58 ± 2.85% after 14 days (*p* > 0.05). These values are consistent with the storage-dependent changes in apparent EE discussed in Section 3.4.1.

Additional gastric-stage release remained low, ranging from 1.17% to 2.67%. Although the overall analysis indicated that the effect of storage duration differed between DEs and CLBs, no significant differences (*p* > 0.05) were detected among the individual formulation means. This indicates limited additional release during gastric incubation rather than an absence of gallic acid in the external phase at the beginning of digestion. Among the individually loaded formulations, SIF-triggered release of gallic acid was statistically similar (*p* > 0.05) for all formulations evaluated after storage for 48 h but was statistically higher (*p* < 0.05) for CLBs when evaluated after 14 days of storage. For samples stored for 14 days, SIF-triggered release was 50.04 ± 4.42% for CLBs, while it was only 24.78 ± 3.43% and 33.66 ± 4.32% respectively for SFO- and decane-based DEs. This difference was attributed to a higher fraction of pre-release during storage in DEs. The SIF-induced triggered release could be associated with the migration and coalescence of W_1_ droplets into W_2_, following the exposure to SIF. The combined effects of NaTC, salts, enzymes, and pH conditions may contribute to interfacial destabilization and facilitate release of the encapsulated gallic acid. These findings are consistent with previous reports for DEs, which show immediate migration of internal droplets upon SIF exposure.^34,51^ CLBs exhibited a very rapid release of W_1_, as shown in Figure 5c and Multimedia component 1. Following triggered release, a substantial fraction (Supporting Table S1) of gallic acid was gradually released between 2.25–6 h in SIF. By the end of the 6 h digestion period, about 0.99–4.27% of gallic acid remained unreleased according to the analytical mass balance.

Overall, CLBs showed consistent release levels at different stages between 48 h and 14 days, while pre-release in DEs increased with storage time and subsequently reduced the intestinal release. Although this study was carried out for CLBs stored in precursor form (as FDDE) prior to rehydration, previous study with comparable-sized CLBs reported that over ∼84% of hydrophilic actives remained encapsulated even after 48 h of rehydration.^19^ A related triple-emulsion CLB study reported hydrophilic-component entrapment of 70–82% and appreciable stability during 2 weeks of rehydration.^21^ These reductions were suggested to be due to rupturing of some unstable fractions during equilibration, with no further release at the later storage period.^19,21^

#### 3.5.2 Curcumin

Storage duration did not significantly affect (*p* > 0.05) any curcumin release fraction. The pre-release fraction ranged from 2.45 ± 0.55% to 3.93 ± 0.51%, while only an additional 0.29 ± 0.19 to 0.43 ± 0.25% was released during gastric incubation with no statistical difference (*p* > 0.05) across formulations with individual encapsulation (Supporting Table S2). The major release response occurred following exposure to SIF. SFO-based DEs showed the highest triggered release (14.26 ± 2.82% and 14.14 ± 2.75% for sample stored for 48 h and 14 days, respectively). In contrast, CLBs showed the lowest release (5.29 ± 1.36% and 5.37 ± 1.20% for sample stored for 48 h and 14 days, respectively). However, the subsequent release during continued incubation in SIF between 2.25–6 h was significantly higher (*p* < 0.05) for CLBs, reaching 33.24 ± 3.39% after 48 h and 33.58 ± 3.53% after 14 days, compared with 17.64 ± 3.24% and 17.91 ± 3.42% in SFO-based DEs and 15.35 ± 2.06% and 15.59 ± 2.18% in decane-based DEs. These results indicate that SFO-based DEs produced a greater immediate response to SIF, whereas CLBs exhibited a smaller initial response followed by greater release during the subsequent intestinal-incubation period. Such release in response to SIF could be associated with progressive interfacial destabilization in the presence of NaTC,^45–47^ and the intestinal environment.^2,9^ Despite the greater subsequent release from CLBs, more than 50% of curcumin remained unreleased after 6 h. The unreleased fraction in CLBs was significantly lower (*p* < 0.05) (58.01 ± 3.89% after 48 h and 57.60 ± 3.92% after 14 days) than that in decane-based DEs (73.23 ± 1.84% for 48 h storage and 72.80 ± 2.08% for 14 days storage) but statistically similar (*p* > 0.05) to that in SFO-based DEs (64.31 ± 1.89% for 48 h of storage and 63.68 ± 2.77% for 14 days of storage). Curcumin release following SIF exposure may involve interfacial destabilization, bile-mediated solubilization, and dissolution or mobilization of particulate curcumin. The relative contributions of these processes were not independently measured. The limited overall release is consistent with the low aqueous solubility of curcumin and its continued association within the carrier structures.^52^

#### 3.5.3 Co-Encapsulation

Co-encapsulation did not significantly affect (*p* > 0.05) the pre-release of gallic acid after 48 h of storage (p < 0.05). However, after 14 days of storage, it significantly reduced gallic acid pre-release in DEs (*p* < 0.05). In SFO-based DEs, the fraction decreased from 62.47 ± 3.63% in the individually loaded system to 45.25 ± 3.80% in the co-encapsulated system, whilst in decane-based DEs, it decreased from 38.20 ± 2.19% to 29.93 ± 3.42%. No corresponding effect was observed in CLBs, for which the pre-release fraction remained ∼11.5% regardless of loading mode.

Co-encapsulation did not significantly affect (*p* > 0.05) gastric-stage gallic acid release. For curcumin, co-encapsulation produced a small but statistically significant overall increase in gastric-stage release (*p* < 0.05); however, the released fraction remained below approximately 1.1% in every formulation. In DEs stored for 48 h, co-encapsulation numerically reduced (*p* > 0.05) the SIF-triggered gallic acid fraction and significantly increased (*p* < 0.05) subsequent SIF release. For SFO-based DEs stored for 14 days, co-encapsulation significantly increased (*p* < 0.05) the SIF-triggered fraction, whereas the increase in subsequent SIF release was not statistically significant (*p* > 0.05). In contrast, in CLBs, asignificant reduction (*p* < 0.05) in SIF-triggered release and a significant increase (*p* < 0.05) in subsequent intestinal-stage release were observed. For 48 h storage samples, SIF-triggered fraction decreased from 49.35 ± 4.83% to 36.80 ± 3.83%, while the subsequent SIF release increased from 33.47 ± 2.60% to 43.69 ± 4.87%. Similar changes were observed for samples stored for14 days, whereby SIF-triggered fraction decreased from 50.04 ± 4.42% to 37.11 ± 3.88%, while the subsequent intestinal-stage release increased from 33.80 ± 2.63% to 44.07 ± 3.66%. Thus, co-encapsulation shifted part of the gallic acid release from the immediate response following SIF addition to the later intestinal-incubation period. However, this shift did not produce significant change (*p* > 0.05) in the unreleased gallic acid fraction within CLBs.

Co-encapsulation did not significantly affect the pre-release, gastric-stage release, or immediate SIF-triggered release of curcumin (*p* > 0.05). However, it significantly increased (*p* < 0.05) subsequent intestinal-stage release. The increase was greatest in CLBs, rising from 33.24 ± 3.39% to 42.31 ± 3.16% after 48 h of storage and from 33.58 ± 3.53% to 43.31 ± 3.31% after 14 days (*p* < 0.05). Consequently, the unreleased curcumin fraction decreased from 58.01 ± 3.89% to 47.29 ± 3.60% after 48 h and from 57.60 ± 3.92% to 46.20 ± 3.76% after 14 days (Supporting Table S2). Co-encapsulation also reduced the mean unreleased curcumin fraction in the DEs by ∼5.7–6.0 percentage points, but these differences were not statistically significant (*p* > 0.05). The mechanism underlying the effects of co-encapsulation was not resolved. Because the formulations were otherwise identical in composition, processing conditions, and particle size, the observed differences are most reasonably associated with the presence of the co-encapsulated active compound. The presence of gallic acid may have altered the solubilization, partitioning, or release behaviour of curcumin, while curcumin may likewise have influenced the retention and release of gallic acid. Co-encapsulation-dependent changes in bioactive retention and release have been observed in other delivery matrices.^28,53^ However, no spectroscopic analysis, partition-coefficient measurements, solubility measurements, or other molecular interaction studies were performed. Therefore, direct gallic acid–curcumin complexation cannot be confirmed, and the observed effects are interpreted as unresolved interactions arising from co-encapsulation.

Collectively, these results show that DEs progressively accumulated gallic acid in W_2_ during storage, whereas the dry-stored FDDE/CLB workflow preserved a greater carrier-associated fraction of gallic acid. Both systems showed limited additional release during gastric incubation and pronounced gallic acid release following SIF exposure, while curcumin release was lower and more formulation dependent. Co-encapsulation improved gallic acid retention in DEs and modified intestinal release, particularly in CLBs. These findings reflect the combined effects of intermediate-phase state, freeze-drying, dry storage, rehydration, and analyte dispersion state. Because these effects could not be separated and several interfacial and molecular mechanisms were not directly characterized, the proposed explanations remain interpretative. Nevertheless, the FDDE/CLB workflow maintained gallic acid retention over 3 weeks, enabled on-demand reconstitution, limited gastric-stage release, and supported substantial intestinal-stage release. Thus, DE-templated CLBs represent a promising dry-state platform for the storage and gastrointestinal delivery of compartmentalized bioactives, particularly hydrophilic compounds susceptible to migration in conventional DEs; co-encapsulation additionally enhanced subsequent intestinal curcumin release and reduced its unreleased fraction in CLBs.

## 4 Conclusions

This study provides a comparative assessment of co-encapsulation and in vitro gastrointestinal release of a hydrophilic polyphenol (gallic acid) and a hydrophobic bioactive (curcumin) in two related hierarchical colloidal systems: conventional W_1_/O/W_2_ DEs and templated W_1_/A/W_2_ CLBs. Both systems achieved high apparent encapsulation efficiencies and effectively limited additional release under simulated gastric conditions, while allowing enhanced release under simulated intestinal conditions. Distinct differences in encapsulation stability and intestinal release profiles were observed between DEs and CLBs, particularly for gallic acid, with the dry-stored FDDE/CLB workflow exhibiting improved retention during 3 weeks of storage and substantial intestinal-stage release. Co-encapsulation improved gallic acid retention in DEs and enhanced intestinal curcumin release from CLBs, thereby reducing the unreleased curcumin fraction. These differences may be associated with the transformation of the intermediate phase and the freeze-drying and rehydration steps required to generate CLBs. Consequently, the observed behaviors reflect the combined influence of intermediate-phase state, interfacial restructuring, and storage history rather than the isolated effect of replacing a liquid oil phase with air. Within these constraints, the paired DE–CLB systems constitute a useful comparative model platform for examining associations between phase connectivity, processing history, retention, and gastrointestinal release in hierarchical colloidal carriers. The results highlight the importance of considering both colloidal architecture and processing pathways when interpreting digestion-responsive release behavior in multi-compartment delivery systems. From a formulation perspective, DE-templated CLBs represent a promising dry-state, on-demand reconstitutable platform that may facilitate storage and handling while supporting the gastrointestinal delivery of compartmentalized bioactives, particularly hydrophilic compounds susceptible to migration in conventional DEs.

## Supplementary Material





## Data Availability

The data underlying this study are available in the published article and its Supporting Information.

## Notes

Core-laden bubbles in SIF_10 × speed (MP4)
The amount of gallic acid (%) and curcumin (%) that was pre-released (0 h), released in simulated gastric fluid (SGF-released, 2 h), triggered release upon addition of simulated intestinal fluid (SIF-triggered release; 2–2.25 h), subsequent intestinal-stage release in SIF (SIF-subsequent release; 2.25–6 h), and unreleased when encapsulated or co-encapsulated in double emulsions (DEs) and core-laden bubbles (CLBs) upon evaluation after 48 h and 14 days of storage (PDF)
