# Supplementary material for: Co-Encapsulation and In Vitro Gastrointestinal Release of Gallic Acid and Curcumin: A Comparison between Double Emulsions and Templated Core-Laden Bubble Systems
Source: ACS Omega. 2026 Sep 10;11(37):55051–62. doi: 10.1021/acsomega.6c01415 (PMC13613875; doi:10.1021/acsomega.6c01415)
Supplement: Supplementary file 1 [file ao6c01415_si_002.pdf]

Co-encapsulation and in vitro gastrointestinal release of gallic acid and curcumin: a comparison between double emulsions and templated core-laden bubble systems

Anuj Niroula[1], Rabia Zia[2,3], Mutamed Ayyash[1], Akmal Nazir[1,*]

[1] Department of Food Science, College of Agriculture and Veterinary Medicine, United Arab Emirates University, Al Ain, 15551, United Arab Emirates

[2] Department of Pharmaceutics, Utrecht Institute for Pharmaceutical Sciences, Utrecht University, Utrecht, the Netherlands

[3] Department of Pharmacology and Therapeutics, College of Medicine and Health Sciences, United Arab Emirates University, Al Ain, 15551, United Arab Emirates

***Correspondence: akmal.nazir@uaeu.ac.ae (Nazir, A)**

Supp. Table S1 The amount of gallic acid (%) that was pre-released (0 h), released in simulated gastric fluid (SGF-released, 2 h), triggered release upon addition of simulated intestinal fluid (SIF-triggered release; 2-2.25 h), subsequent intestinal-stage release in SIF (SIF-subsequent release; 2.25-6 h), and unreleased when encapsulated or co-encapsulated in double emulsions (DEs) and core-laden bubbles (CLBs) upon evaluation after 48 h and 14 days of storage. The same small letter alphabets in subscript indicate no significant ($p > 0.05$) difference amongst the sample means for each column.

System	Intermediate phase	Active component	Pre-released (%)	SGF-released (%)	SIF-triggered release (%)	SIF-subsequent release (%)	Unreleased within 6 h (%)
**After 48 h**							
DE	SFO	Encapsulation	18.02 ± 2.78 ^{d}	1.17 ± 0.26 ^{a}	55.62 ± 4.75 ^{a}	21.89 ± 2.41 ^{de}	3.30 ± 1.36 ^{ab}
DE	Decane	Encapsulation	12.48 ± 1.76 ^{d}	1.95 ± 0.23 ^{a}	54.63 ± 4.62 ^{a}	26.66 ± 2.67 ^{cd}	4.27 ± 1.60 ^{ab}
CLBs	Air	Encapsulation	11.52 ± 2.40 ^{d}	1.78 ± 0.82 ^{a}	49.35 ± 4.83 ^{ab}	33.47 ± 2.60 ^{bc}	3.89 ± 2.60 ^{ab}
DE	SFO	Co-encapsulation	16.33 ± 2.12 ^{d}	1.48 ± 0.84 ^{a}	45.28 ± 4.40 ^{abc}	31.90 ± 2.36 ^{bc}	5.01 ± 1.84 ^{ab}
DE	Decane	Co-encapsulation	11.51 ± 2.02 ^{d}	1.44 ± 0.68 ^{a}	44.36 ± 3.01 ^{abc}	36.96 ± 2.05 ^{ab}	5.71 ± 1.44 ^{ab}
CLBs	Air	Co-encapsulation	11.64 ± 2.72 ^{d}	1.54 ± 0.38 ^{a}	36.80 ± 3.83 ^{cd}	43.69 ± 4.87 ^{a}	6.34 ± 1.19 ^{a}
**After 14 days**							
DE	SFO	Encapsulation	62.47 ± 3.63 ^{a}	2.52 ± 0.68 ^{a}	24.78 ± 3.43 ^{d}	9.24 ± 2.99 ^{f}	0.99 ± 0.44 ^{b}
DE	Decane	Encapsulation	38.20 ± 2.19 ^{b}	2.66 ± 0.82 ^{a}	33.66 ± 4.32 ^{cd}	22.17 ± 2.85 ^{de}	3.31 ± 1.68 ^{ab}
CLBs	Air	Encapsulation	11.58 ± 2.85 ^{d}	1.65 ± 0.37 ^{a}	50.04 ± 4.42 ^{a}	33.80 ± 2.63 ^{bc}	2.93 ± 2.63 ^{ab}
DE	SFO	Co-encapsulation	45.25 ± 3.80 ^{b}	2.03 ± 0.36 ^{a}	37.57 ± 4.39 ^{bc}	13.95 ± 2.54 ^{ef}	1.19 ± 0.24 ^{b}
DE	Decane	Co-encapsulation	29.93 ± 3.42 ^{c}	2.67 ± 0.69 ^{a}	34.59 ± 3.94 ^{cd}	28.41 ± 2.33 ^{bcd}	4.40 ± 0.92 ^{ab}
CLBs	Air	Co-encapsulation	11.75 ± 2.75 ^{d}	1.55 ± 0.38 ^{a}	37.11 ± 3.88 ^{bc}	44.07 ± 3.66 ^{a}	5.51 ± 1.62 ^{ab}

Supp. Table S2 The amount of curcumin (%) that was pre-released (0 h), released in simulated gastric fluid (SGF-released, 2 h), triggered release upon addition of simulated intestinal fluid (SIF-triggered release; 2-2.25 h), subsequent intestinal-stage release in SIF (SIF-subsequent release; 2.25-6 h), and unreleased when encapsulated or co-encapsulated in double emulsions (DEs) and core-laden bubbles (CLBs) upon evaluation after 48 h and 14 days of storage. The same small letter alphabets in subscript indicate no significant ($p > 0.05$) difference amongst the sample means for each column.

System	Intermediate phase	Active component	Pre-released (%)	SGF-released (%)	SIF-triggered release (%)	SIF-subsequent release (%)	Unreleased within 6 h (%)
**After 48 h**							
DE	SFO	Encapsulation	3.45 ± 0.37 ^{a}	0.34 ± 0.22 ^{a}	14.26 ± 2.82 ^{ab}	17.64 ± 3.24 ^{c}	64.31 ± 1.89 ^{bcd}
DE	Decane	Encapsulation	2.45 ± 0.55 ^{a}	0.43 ± 0.25 ^{a}	8.54 ± 2.35 ^{bc}	15.35 ± 2.06 ^{c}	73.23 ± 1.84 ^{a}
CLBs	Air	Encapsulation	3.06 ± 0.64 ^{a}	0.41 ± 0.28 ^{a}	5.29 ± 1.36 ^{c}	33.24 ± 3.39 ^{b}	58.01 ± 3.89 ^{d}
DE	SFO	Co-encapsulation	3.75 ± 0.65 ^{a}	0.89 ± 0.22 ^{a}	17.41 ± 2.41 ^{a}	19.56 ± 1.97 ^{c}	58.39 ± 2.03 ^{d}
DE	Decane	Co-encapsulation	2.94 ± 0.83 ^{a}	0.45 ± 0.24 ^{a}	10.48 ± 2.37 ^{bc}	18.62 ± 2.69 ^{c}	67.50 ± 2.87 ^{abc}
CLBs	Air	Co-encapsulation	3.22 ± 0.39 ^{a}	0.95 ± 0.31 ^{a}	6.24 ± 1.93 ^{c}	42.31 ± 3.16 ^{a}	47.29 ± 3.60 ^{e}
**After 14 days**							
DE	SFO	Encapsulation	3.93 ± 0.51 ^{a}	0.35 ± 0.22 ^{a}	14.14 ± 2.75 ^{ab}	17.91 ± 3.42 ^{c}	63.68 ± 2.77 ^{cd}
DE	Decane	Encapsulation	2.50 ± 0.56 ^{a}	0.29 ± 0.19 ^{a}	8.83 ± 2.22 ^{bc}	15.59 ± 2.18 ^{c}	72.80 ± 2.08 ^{ab}
CLBs	Air	Encapsulation	3.12 ± 0.45 ^{a}	0.43 ± 0.18 ^{a}	5.37 ± 1.20 ^{c}	33.58 ± 3.53 ^{b}	57.60 ± 3.92 ^{d}
DE	SFO	Co-encapsulation	3.83 ± 0.65 ^{a}	0.91 ± 0.30 ^{a}	17.86 ± 2.59 ^{a}	19.67 ± 2.27 ^{c}	57.73 ± 2.49 ^{d}
DE	Decane	Co-encapsulation	3.00 ± 0.85 ^{a}	0.46 ± 0.32 ^{a}	10.69 ± 2.28 ^{bc}	18.86 ± 2.43 ^{c}	66.99 ± 2.34 ^{abc}
CLBs	Air	Co-encapsulation	3.25 ± 0.40 ^{a}	1.04 ± 0.36 ^{a}	6.19 ± 1.78 ^{c}	43.31 ± 3.31 ^{a}	46.20 ± 3.76 ^{e}
